# Measures of predator diet alone may underestimate the collective impact on prey: Common buzzard *Buteo buteo* consumption of economically important red grouse *Lagopus lagopus scotica*

**DOI:** 10.1371/journal.pone.0221404

**Published:** 2019-08-20

**Authors:** Richard M. Francksen, Nicholas J. Aebischer, Sonja C. Ludwig, David Baines, Mark J. Whittingham

**Affiliations:** 1 School of Natural and Environmental Sciences, Newcastle University, Newcastle-Upon-Tyne, England, United Kingdom; 2 Game and Wildlife Conservation Trust, Burgate Manor, Fordingbridge, Hampshire, England, United Kingdom; 3 Game and Wildlife Conservation Trust, Coach House, Eggleston, Barnard Castle, Co. Durham, England, United Kingdom; University of Southern Queensland, AUSTRALIA

## Abstract

Human-wildlife conflicts often centre on economic loss caused by wildlife. Yet despite being a major issue for land-managers, estimating total prey losses to predation can be difficult. Estimating impacts of protected wildlife on economically important prey can also help management decisions to be evidence-led. The recovery in population and range of common buzzards *Buteo buteo* in Britain has brought them into conflict with some gamebird interests. However, the magnitude of any impact is poorly understood. We used bioenergetics models that combine measures of buzzard abundance from field surveys with diets assessed by using cameras at nests, prey remains and pellet analysis, to estimate their impact on red grouse *Lagopus lagopus scotica* on a large (115 km^2^) moor managed for red grouse shooting in Scotland. Whilst grouse consumption by individual buzzards was lower than previous estimates for other raptor species present on our study site, total consumption could be greater given an estimated 55–73 buzzards were present on the study site year-round. Averaging across diet assessment methods, consumption models estimated that during each of three breeding seasons (April-July 2011–2013), the buzzards foraging on our study site consumed 73–141 adult grouse and 77–185 chicks (depending on year). This represented 5–11% of adult grouse present in April (22–67% of estimated adult mortality) and 2–5% of chicks that hatched (3–9% of estimated chick mortality). During two non-breeding seasons (August-March), consumption models using pellet analysis estimated that buzzards ate a total of 242–400 grouse, equivalent to 7–11% of those present at the start of August and 14–33% of estimated grouse mortality during the non-breeding season. Buzzard consumption of grouse has the potential to lead to non-trivial economic loss to grouse managers, but only if buzzards predated the grouse they ate, and if grouse mortality is additive to other causes.

## Introduction

Quantifying the impact of predation on prey numbers can be a complex and controversial issue in ecology [1], but necessary when considering management solutions that deliver both economic and conservation objectives [2,3]. Generalist predators can drive prey population dynamics through their ability to switch between available prey resources [4,5]. Predator removal experiments can provide rigorous estimates of predator impact on prey [6,7]. However, when this approach is not possible owing to legal protection of predators, estimates of predator diet and abundance within bioenergetics models can be useful when estimating prey consumption levels and local impacts on prey [3,8–10].

Population recovery of several raptor species in Britain [11] has intensified concern over their impact on gamebirds [7,12]. The common buzzard *Buteo buteo* (herein ‘buzzard’) is a medium-sized generalist raptor. Following reductions in persecution and the use of organo-chlorine pesticides, and increases in some prey groups [13,14], the breeding range of buzzards has increased by an estimated 81% over the last 40 years, and the winter range has increased by 74% in the last 30 years[11]. This makes buzzards the most abundant diurnal raptor in Britain, with a recent population estimate of 56,000–77,000 breeding pairs [15]. Buzzards preferred prey are field voles *Microtus agrestis* and European rabbits *Oryctolagus cuniculus*, but they also eat pheasants *Phasianus colchicus* released for shooting [16,17] and wild red grouse *Lagopus lagopus scotica* [18–20].

Red grouse (herein 'grouse') are considered to be an economically important game bird in parts of the U.K. uplands, with the average cost of shooting a brace (two birds) estimated at £150 (GBP) and the management of grouse providing a source of rural employment [21]. Heather moorland managed principally for grouse shooting represents 5–15% of the U.K. uplands, and 20–40% of all heather-dominated moorland [22]. A long-term decline in grouse numbers in Britain has been largely associated with declines in heather-dominated moorland, upland afforestation, reductions in gamekeepers and associated increases in generalist predators including red fox *Vulpes vulpes* and corvids *Corvus spp*. [23–25]. Predation by raptors, particularly hen harriers *Circus cyaneus* and peregrines *Falco peregrinus*, has been sufficient to reduce post-breeding grouse numbers to levels incompatible with continued driven shooting [26]. Driven grouse shooting involves driving grouse towards a line of paying hunters who shoot the grouse as they fly past. This requires higher grouse densities, and can generate substantially greater revenue, than alternative forms of grouse shooting [21]. The potential for buzzard predation to impact on grouse abundance has hitherto not been estimated, but is necessary to ensure that management decisions are based on sound evidence. This evidence can help to target interventions and avoid expensive and ineffective measures aimed at reducing the impact of predation [2,27–29].This paper seeks to quantify the consumption of grouse by buzzards on a Scottish moor managed for grouse by combining estimates of buzzard diet with bioenergetics and estimates of buzzard abundance. Methods of assessing raptor diet carry inherent sources of bias [30,31], which, for buzzards, can also vary between years as diet changes with prey availability [19]. To test the biases associated with detection of large (grouse) and small (vole) prey in buzzard diet, we conducted a controlled feeding trial using four captive buzzards housed at falconry display centres. Relying on predator diet data alone, without combining with information on predation rates and densities of both predators and prey, can misrepresent the impact of predation on prey[3,8–10]. Here we use estimates of buzzard diet and abundance in bioenergetics and consumption models to estimate total prey consumption, which we then compare with estimates of grouse abundance to assess the potential impact of buzzards under a range of scenarios.

## Materials and methods

### Measuring buzzard diet

We studied buzzard diet and foraging behaviour during 2011–2015 at Langholm Moor (55.1–55.3°N, 3.0–2.8°W), a 115-km^2^ mosaic of heather and acid grass moorland in south-west Scotland. Here moorland management was undertaken by a team of five gamekeepers, which included burning and cutting of heather, and lethal control of generalist predators (primarily red fox *Vulpes vulpes*, carrion crow *Corvus corone*, stoat *Mustela ermine* and weasel *Mustela nivalis*). Gamekeepers were employed as part of a partnership project that began in 2008,with the objective of restoring post-breeding grouse densities to a level sufficient to resume driven commercial sport shooting, while supporting a viable population of hen harriers. All raptor species, including buzzards, were strictly protected and monitored as part of the wider project.

Thirty-two successful buzzard nests from 36 territories were studied throughout the 50-day nestling period (11 in 2011, 10 in 2012, 11 in 2013). Prey delivery to chicks was studied using three methods: nest cameras, prey remains and pellets (for more details of methods see [19,20]). Nest cameras were attached to a branch within 1–2 m of the nest to allow the entire nest platform to be observed. We aimed to minimise disturbance by installing cameras after hatching was completed, and as quickly as possible during calm weather [32]. No study nests were abandoned following installation of cameras. Motion-triggered video images of 1–5 minutes in duration were stored on a recording unit (model: Mini HDVR LS-H720) before being analysed. Images were collected from each buzzard nest for at least three days in each of three nestling periods: < one week, one to four weeks and > four weeks old. Overall, 2,320 hours of footage were collected (80 ± 15 se hours nest^-1^), yielding 869 prey deliveries (27 ± 3 nest^-1^). Searches for prey remains and regurgitated pellets were conducted inside the same nests and within a 50-m radius of them at the end of each camera recording period, and again during the first week post-fledging, yielding 486 prey remains (15 ± 1 nest^-1^) and 220 pellets (7 ± 1 nest^-1^). Analysis of pellets yielded 582 prey items, which were assumed to represent one individual prey animal in each case, unless shown otherwise [31]. Buzzard diet in two winters (October-February) was estimated from pellets collected at 23 roosts sites in 2013/14 and 21 roosts in 2014/15 (19 in both years). Roost sites were searched fortnightly for pellets, yielding 409 pellets containing 1,107 prey items in 2013/14, and 355 pellets with 993 prey items in 2014/15.

### Controlled feeding trial

We conducted 30 individual feeding trials, which involved presenting a total of 60 voles, 18 grouse and five pheasants to four captive buzzards. Trials began only after pellet production from a meal pre-dating the experiment indicated an empty crop. In each trial, a buzzard was presented with a gamebird carcass (either grouse or pheasant). Once the buzzard had stopped feeding, between one and four voles (mean 2.2 ± 0.2) were offered by hand. The amount of gamebirds eaten, as a proportion of all food eaten during the trial, was then calculated by weighing any uneaten food and subtracting it from the weight of food provided at the start of the trial. Buzzards typically produce one pellet per day, but may delay pellet production for up to three days [33]. If no pellet was produced within 24 hours of the trial, the trial would continue the next day, with the food items summed across trial-days. Two measures of gamebird and vole detectability in pellets were calculated for each buzzard: (i) ‘presence detection rate’–the percentage of pellets where the prey type was detected relative to the number of pellets produced following a meal containing any amount of that prey; and (ii) ‘item detection rate’–the number of prey items detectable in pellets as a percentage of the actual number of items of that prey consumed. Measures from each of the four buzzards were averaged.

### Estimation of buzzard numbers

In 2011–2013, all active nests within the study site were located (see [19]). In addition, we included a 1-km buffer zone around the site, constituting a further 50 km^2^, to include additional breeding buzzards that, based on circular home ranges of radius 1 km, hunted within the study site [20]. We assumed that these buzzard nests occurred on average 0.52 km into the 1-km buffer zone (the distance that split the buffer zone into two concentric rings of equal area). As a circular buzzard territory of radius 1 km covered 3.14 km^2^, we calculated that an average of 0.55 km^2^ of territory (i.e. 17.5%) overlapped with the study area, using formulae for the area of circle-circle intersections [34]. The estimated number of additional buzzards in the buffer zone was then multiplied by 0.175 to give an equivalent number of individuals that would be expected to hunt full-time within the study area, for inclusion in impact calculations.

Buzzard brood size averaged 1.7 chicks up to the age of 25 days, and 1.6 chicks from 26 to 50 days. Non-breeders, either juveniles, sub-adults or adults without breeding territories, can represent a considerable proportion of a raptor population [14] and need to be included when estimating predator impact [35]. The only estimate for the proportion of non-breeding buzzards in Britain is based on ringing data and suggests that 36% of buzzards alive in spring are breeders [36], an estimate that we use here.

Numbers of buzzards present in winter 2013/14 and 2014/15 were estimated using a mark–resighting method involving 50 fledglings and 35 juveniles or sub-adults caught and individually wing-tagged between June 2012 and November 2014. Observations of tagged birds seen during three-hour vantage point surveys, conducted monthly between November and March on 12 (2013/14) or 8 (2014/15) 2-km^2^ areas of the moor, were combined with incidental sightings to estimate the number of marked individuals present at the start of each month of two winters. An estimated number of buzzards using the study area was obtained monthly using a Lincoln-Petersen Index [37] of the ratio of tagged to un-tagged birds from the re-sighting data that month in relation to the number of tagged individuals present at the start of the month. Monthly pairs of observations (November & December, December & January, January & February) were compared in this way to reduce the possible effect of movements on and off the site on estimates of numbers present. By doing so, we produced three estimates, which were then averaged, for each of two winters, 2013/14 and 2014/15. Project methods were prospectively approved by, and conducted under license from, Scottish Natural Heritage (Bird License number 65053).

### Bioenergetics model

Bioenergetic and prey consumption models were constructed to estimate total grouse consumption by buzzards. Models incorporated equations taken from published literature (Table 1) and used field estimates of buzzard diet and abundance, averaged within year, while other input parameters were taken from published sources (Table 2). Models estimated the daily energy requirements of a buzzard, depending on age, sex and breeding status, and converted these into daily and total food requirements. By combining bioenergetics estimates with the proportion of buzzard diet consisting of grouse, we estimated the number of grouse eaten per buzzard. This was then multiplied by buzzard abundance estimates for each class and by the corresponding foraging period (days), then totalled to estimate grouse consumed by all buzzards in a defined season.

**Table 1:** Calculations used in buzzard bioenergetics and grouse consumption models.

Parameter	Equation	Notes
Age (A) of nestling buzzard (days) [38]	12.8 + 0.1 × P5	P5 is the length of the 5th primary in mm
Mass (M) of nestling buzzard (grams) [This study]	568.4 × log(A) - 1300.5	A is estimated age in days (see above)
Field Metabolic Rate (FMR) of non-breeding adult (kJ/day) [39]	10.5 × M^0.68^	M is mass in grams
FMR of incubating female (kJ/day) [40]	20.8 × M^0.46^	
FMR of chick-rearing adult (kJ/day) [39]	13.8 × M^0.65^	
FMR of nestling (kJ/day) [39]	4.58 × M^0.76^	
Daily Food Requirement (DFR) (grams) [41]	FMR ÷ (energy content of food × (1—moisture content) × Assimilation efficiency)	Energy content of food is in kJ/g, and moisture content and assimilation efficiency are proportions between 0 and 1.
Total Food Requirement (TFR)	FMR x D	D is time in days
Number of grouse eaten [4]	(TFR × PB) ÷ (MMP × 100)	PB is percentage biomass in buzzard diet consisting of grouse; and MMP is mean digestible mass of grouse in grams.

**Table 2:** Average values (± SE) used as parameters in buzzard bioenergetics and grouse consumption models.

		Source
Breeding pairs	13±0	This study
Breeding rate	35.5±6.4%	[36]
Brood size (0–25 days)	1.693±0.058	This study
Brood size (26–50 days)	1.563±0.045	This study
Incubation period	35±2 days	[42]
Nestling period	50±6 days	[32]; this study
Post-fledging period	37±6 days	This study
Winter buzzard numbers (2013/14)	53.8±9.0	This study
Winter buzzard numbers (2014/15)	64.8±10.9	This study
Total summer period	122 days	This study
Total winter period	243 days	This study
Adult female buzzard mass	1000±42 g	[43]
Adult male buzzard mass	780±42 g	[43]
Buzzard chick mass	Adjusted for age*	This study
Adult red grouse mass (mean of sexes)	600±32 g	[43]
Red grouse chick mass (June)	61.3±6.7 g	S. Ludwig, unpublished data
Ingestion rate	75.0±2.3%	[44]
Food assimilation efficiency	82.0±6.6%	[45]
Food moisture content	72.43±2.90%	[46]
Food energy content	23.18±2.32 kJ/g	[46]

* Buzzard chick mass calculated using equations in Table 1.

The daily energy requirement was taken as the ‘Field Metabolic Rate’ (FMR), which measures the energy requirement of a free-living animal behaving normally in its natural habitat [39]. Since body mass and phylogeny account for over 93% of variation in FMR, we estimated FMR for individual buzzards depending on age and sex using allometric equations [39,40]. Breeding male buzzards provision their incubating females [32], so the equation for ‘chick-rearing adult’ was used for calculating FMR for breeding male buzzards during both incubation and chick-rearing periods. Since adult females are approximately 20% heavier than males [43], FMR was calculated separately for each sex.

The age of 58 buzzard nestlings was estimated from the length of the fifth primary feather [38]. This, together with weight, were used to derive an empirical relationship between mass and estimated age. Daily and total FMR for nestlings could then be calculated as for adults. Nestlings were not sexed, instead sex ratios were assumed equal, and were considered full-grown at fledging [32].

Daily Food Requirements (DFR) of individuals were estimated from FMR values (according to age, sex of adults and breeding status) using aggregate food energy and moisture content values for vertebrate prey [46] and assimilation efficiency for *Accipitriformes* [45] (Tables 1 and 2). Total Food Requirement (TFR) was obtained by multiplying DFR by the appropriate time in days. The breeding period averaged 122 days, which included the buzzard incubation period (35 days) beginning in early-April [42], the nestling period (50 days) [32] and 37 days during which all fledglings were assumed to remain on-site [47]. The non-breeding period averaged 243 days, during which time all buzzards were considered as non-breeding adults for the purposes of calculations.

To estimate the number of grouse consumed by an individual buzzard, we used the calculations of Korpimäki and Norrdahl [4]. First, buzzard prey consumption in summer and winter estimated from nest cameras, prey remains and pellets was converted to biomass by summing the weights of all prey items. Mammal weights were from Aulagnier *et al*. [48] and Salamolard *et al*. [49], and bird weights from Snow & Perrins [43]. Weight of meadow pipit *Anthus pratensis*, the commonest passerine in the study area [50], was used for unidentified small passerines, that of field vole for unidentified small mammals and that of European rabbit for unidentified *Lagomorph spp*. Weights of invertebrates, amphibians and reptiles were from Salamolard *et al*. [49], Rooney & Montgomery [51] and ARKive [www.arkive.org]. TFR was multiplied by the proportion of biomass consisting of grouse to estimate total biomass of grouse consumed by an individual buzzard, with separate estimates for adult grouse and chicks by each dietary assessment method and in each breeding and non-breeding period.

Kenward *et al*. [44] estimated that 75% of a pheasant’s mass was digestible by goshawks *Accipiter gentilis*, a value subsequently used by Tornberg *et al*. [52] when considering black grouse *Lyrurus tetrix* consumption by buzzards. We used the same value for red grouse consumption by buzzards, hence 75% of a 600-g grouse (450 g) was assumed to be available for consumption. We used a mean grouse chick weight at 15 days old of 61.3 g [S. Ludwig, unpublished data], with no adjustment for indigestible parts because chicks were observed to be eaten whole and the indigestible portion of a g**r**ouse chick would be small [53]. Dividing total grouse biomass consumed (g) by 450 (adults) and 61 (chicks) gave estimates of the numbers of each that had been eaten by buzzards.

### Estimating grouse abundance

The estimated number of grouse eaten by buzzards was compared to the estimated number of grouse present from surveys, and to the estimated numbers of grouse lost between consecutive surveys. Pre-breeding grouse were counted annually in spring (March/early April) along 18 transects (mean length 2.0 km ± 0.2) and within 10 50-ha blocks, and repeated post-breeding in July, using a pointer dog. Distance corrections were applied by recording the perpendicular distance from the transect to each grouse encounter position, before calculating an Effective Strip Width (ESW) using the programme DISTANCE 6.0 [54]. The number of birds observed (adults in spring, young and adults in July) was divided by the area searched (transect length x 2 x ESW) to give a mean grouse density per km^2^. This value was then multiplied by the amount of heather habitat capable of supporting grouse, estimated as 30 km^2^ [29], to estimate total grouse on the moor. Langholm Moor is surrounded by habitat generally unsuitable to red grouse, hence immigration and emigration was likely to be negligible [55].

To estimate the number of grouse chicks available to be eaten and the number of grouse chicks lost between hatching and the July counts in each of the years 2011, 2012 and 2013, we evaluated the number of grouse chicks that hatched following Thirgood *et al*. [26]. Using female grouse caught in winter at night and fitted with necklace-mounted radio-transmitters (Holohil RI-2DM), we monitored 15–23 females annually during breeding and assessed brood size at hatch, counting nest loss as broods of zero unless a second clutch was successful. We calculated July brood size from count data, including females without chicks as broods of zero. Then we used the following formula to calculate chick abundance at hatching: Chick abundance at hatch = Brood size at hatch / July brood size x July number of young.

### Estimation of confidence limits

Confidence limits (95%) were estimated by first recording standard errors associated with all parameters measured, both empirically and from the literature. We then calculated standard errors of products and quotients using Taylor series linearization [37] for each stage of the modelling process and its outcome. This process allowed us to calculate the error introduced at each stage of the estimation process and to calculate approximate 95% confidence limits around the estimates of grouse consumption by buzzards.

## Results

### Feeding trial

Results from 30 feeding trials with four buzzards (S1 Table) showed that, in three feeding trials, buzzards ate only from the gamebird carcass and refused voles, whilst in seven trials buzzards ate only voles and refused gamebird. In the remaining 20 trials, both gamebird and voles were eaten in varying proportions (mean % gamebird 61% ± 5 SE, range: 2–93%). Of the 23 trials in which gamebirds were eaten, gamebirds were identified from feathers in twelve of the resulting 23 pellets. The gamebird presence rate in pellets averaged 52% (± 9 SE) across the four buzzards (range: 33–75%). Presence and item rates were the same for gamebirds, since buzzards ate from only one gamebird for each pellet produced. Of the 27 trials in which voles were eaten, their presence was subsequently detected from fur, teeth, or bones in 26 pellets, with a mean detection rate of 98% (± 2 SE), (range: 92–100%). Of the 60 voles eaten, remains of 30 were found in pellets, with a mean item detection rate of 52% (± 6 SE), (range: 40–67%). Proportion of prey biomass in wild buzzard pellets was adjusted to account for detectability values derived from the controlled feeding trial.

### Grouse occurrence in wild buzzard diet

From camera images at nests, adult grouse averaged between 0% and 2.6% of buzzard prey biomass, whilst chicks averaged between 0% and 0.4%. Using prey remains, adult grouse averaged between 1.7% and 5.1%, with grouse chicks averaging between 0.3% and 0.8%. Using pellet data, which were adjusted for prey detection (see above), adult grouse averaged between 2.2% and 4.2%, and grouse chicks between 0.2% and 0.8%. Pellet analysis from winter roosts showed that grouse averaged 6.6% and 3.4% per winter of total identified prey biomass (Table 3).

**Table 3:** Mean percentage ± SE of total biomass of prey in the diet of an individual buzzard consisting of red grouse adults and chicks. Correction factors were applied to pellet data (see text).

	Adult red grouse	Red grouse chicks
Summer		
*Camera images*		
2011	0.00 ± 0.00	0.00 ± 0.00
2012	2.56 ± 1.43	0.44 ± 0.18
2013	0.98 ± 0.50	0.21 ± 0.14
*Prey remains*		
2011	5.10 ± 2.99	0.83 ± 0.49
2012	3.85 ± 2.17	0.45 ± 0.22
2013	1.67 ± 1.07	0.34 ± 0.14
*Pellet analysis*		
2011	4.18 ± 2.28	0.83 ± 0.47
2012	2.92 ± 1.85	0.55 ± 0.36
2013	2.15 ± 1.00	0.15 ± 0.10
Winter		
Pellet analysis		
2013/14	6.61 ± 2.07	n/a
2014/15	3.39 ± 1.27	n/a

### Estimating buzzard numbers

During 2011–2013, 12 pairs of buzzards bred each year within the study site (0.10 pairs km^-2^). Using this density, an additional five pairs were predicted to occur in the 1-km buffer zone (50 km^2^). The proportion of these additional territories that overlapped the study area was 0.175. Hence, we estimated that a further pair (5 x 0.175) fed full-time in the study area, giving 13 breeding pairs per annum. Each pair produced an average of 1.56 chicks up to the point of fledging (Table 2). Given a breeding rate of 35.5%, 47 non-breeders, assumed of equal sex ratio, were predicted to forage in the study area, giving a total of 73 birds in the breeding season.

During winter 2013/14, 196 buzzard sightings (38 of which were wing-tagged) were made between December and February. Sub-setting the data into pairs of months gave abundance estimates of 71 in December, 40 in January and 54 in February, a mean of 55 (± 9) birds. Equivalent estimates during winter 2014/15 were based on 317 sightings (28 tagged), giving abundance estimates of 69, 81 and 44 for each monthly-pair, and a mean of 65 (± 11).

### Bioenergetics and grouse consumption

Chick-rearing females had 7% higher energy needs than non-breeding females, and chick-rearing by females consumed 2.5 times as much energy as incubation (Table 4). Adult males provisioning for their mates and chicks had 8% higher requirements than non-breeding males. Average FMR and DFR values for chicks were 129% higher in the second half of the nestling period than in the first half as chicks grew. Requirements of nestlings formed 5% of the total food requirement of all buzzards during the summer.

**Table 4:** Estimated average field metabolic rate (FMR) and daily food requirements (DFR) of an individual buzzard at Langholm by age, sex and breeding status. Total FMR and total food requirement (TFR) are calculated for each buzzard class depending on abundance estimates and length of period considered. Values for summer have been pooled across years for brevity.

	FMR (kJ/day/ buzzard)	DFR (g/day/ buzzard)	Period (days)	Individual TFR (kg)	Abundance estimate	Aggregate TFR (kg)
Summer (122 days) (all years)						
Provisioning male	1053.5	201.0	85	17.1	13.0	222
Incubating female	499.0	95.2	35	3.3	13.0	43
Chick-rearing female	1238.5	236.3	50	11.8	13.0	154
Chick in the nest (0–25 days)*	308.7	58.9	25	1.5	22.0	32
Chick in the nest (26–50 days)*	706.0	134.7	25	3.4	20.3	68
Non-breeding male	978.8	186.8	85	15.9	23.6	375
Non-breeding female	1159.3	221.2	85	18.8	23.6	444
Post-fledging period male	978.8	186.8	37	6.9	46.8	323
Post-fledging period female	1159.3	221.2	37	8.2	46.8	383
**Total**						**2044**
Winter (243 days) (2013/14)						
Non-breeding male	978.8	186.8	243	45.4	27.5	1248
Non-breeding female	1159.3	221.2	243	53.8	27.5	1478
**Total**					**55.0**	**2726**
Winter (243 days) (2014/15)						
Non-breeding male	978.8	186.8	243	45.4	32.4	1468
Non-breeding female	1159.3	221.2	243	53.8	32.4	1739
**Total**					**64.7**	**3207**

*Values are averages for each sub-period using growth curves (see Table 1) and adjusted for average brood size.

In all years, the percentage of adult grouse and chicks in buzzard diet was highest when estimated from prey remains. Amongst years, it was highest in 2011 except when estimated from camera images, as no grouse were recorded delivered to buzzard nests in 2011. However, grouse were clearly evident using other methods at the same nest sites (Table 3). Using camera images, we estimated that all buzzards present on our study site ate a total of 116 and 45 adult grouse respectively in 2012 and 2013, representing 9% (95% CI 4–14%) of adult grouse present in spring 2012 and 3% (2–5%) in 2013 (Table 5). They also ate an estimated 147 grouse chicks in 2012 and 67 in 2013, representing 5% (2–8%) and 1% (1–2%) of chicks that hatched (Table 6). Using prey remains, we estimated that buzzards ate 232 adult grouse in 2011, 175 in 2012 and 76 in 2013, representing 19% (8–30%) of grouse present in spring 2011; 13% (6–21%) in 2012 and 6% (2–9%) in 2013. We also estimated that buzzards ate 277 grouse chicks in 2011, 150 in 2012 and 113 in 2013, equivalent to 7% (2–12%), 5% (2–8%) and 2% (1–3%) of chicks that hatched. Using pellets, we estimated that buzzards ate 190 adult grouse in 2011, 133 in 2012 and 98 in 2013, representing 16% (7–24%) of those present in spring 2011, 10% (4–16%) in 2012 and 7% (4–11%) in 2013. Estimates for grouse chicks were 277 in 2011, 184 in 2012 and 50 in 2013 or 7% (3–12%), 6% (2–10%) and 1% (0–2%) of chicks estimated to have hatched.

**Table 5:** Estimated number of adult red grouse consumed by all buzzards at Langholm Moor. Figures are total estimated number of grouse consumed during each of three breeding seasons by three diet assessment methods and during each of two winters using pellets from roost sites. The percentage consumed is evaluated from the numbers of grouse present at the start of the relevant period (breeding (nests) or non-breeding (winter roosts), and to the numbers of grouse lost by the end of it. Diet data were collected from 32 nests (11 in 2011; 10 in 2012; 11 in 2013) and 44 winter roosts (23 in 2013/14; 21 in 2014/15).

Diet assessment method	Year	No. eaten (95% CL)	No. present at start (95% CL)	No. losses (95% CL)	% of present eaten (95% CL)*	% of losses eaten (95% CL)*
Camera images (nests)	2011	0 (0–0)	1224 (1011–1482)	99 (0–373)	0 (0–0)	0 (0–0)
	2012	116 (53–180)	1302 (1164–1458)	210 (10–410)	8.9 (4.0–13.9)	55.4 (0–100)
	2013	45 (22–68)	1392 (1239–1587)	336 (114–558)	3.2 (1.5–4.9)	13.3 (2.2–24.3)
Prey remains (nests)	2011	232 (101–363)	1224 (1011–1482)	99 (0–373)	18.9 (7.6–30.3)	100 (0–100)
	2012	175 (79–271)	1302 (1164–1458)	210 (10–410)	13.4 (5.9–21.0)	83.3 (0–100)
	2013	76 (30–122)	1392 (1239–1587)	336 (114–558)	5.5 (2.1–8.8)	22.6 (2.3–42.9)
Pellets (nests)	2011	190 (88–292)	1224 (1011–1482)	99 (0–373)	15.5 (6.7–24.4)	100 (0–100)
	2012	133 (53–212)	1302 (1164–1458)	210 (10–410)	10.2 (4.0–16.4)	63.2 (0–100)
	2013	98 (51–145)	1392 (1239–1587)	336 (114–558)	7.0 (3.5–10.5)	29.1 (5.3–52.9)
Pellets (winter roosts)	2013/14	400 (148–653)	3675 (3231–4185)	1230 (704–1756)	10.9 (3.9–17.9)	32.6 (7.7–57.4)
	2014/15	242 (72–411)	3627 (3201–4107)	1686 (1181–2191)	6.7 (1.9–11.4)	14.3 (3.4–25.3)

* Negative percentages and ones above 100 were replaced by 0 and 100 respectively.

**Table 6:** Estimated number of red grouse chicks consumed by all buzzards at Langholm Moor. Figures are total estimated number of grouse consumed during each of three breeding seasons by three diet assessment methods. The percentage consumed is evaluated from the numbers of chicks that hatched and the number lost by the end of each breeding season. Diet data were collected from 32 nests (11 in 2011; 10 in 2012; 11 in 2013).

Diet assessment method	Year	No. eaten (95% CL)	No. present at start (95% CL)	No. losses (95% CL)	% of present eaten (95% CL)	% of losses eaten (95% CL)
Camera images (nests)	2011	0 (0–0)	3727 (2400–5053)	2653 (1368–3937)	0 (0–0)	0 (0–0)
	2012	147 (81–213)	3059 (2024–4094)	1697 (713–2681)	4.8 (2.1–7.5)	8.6 (2.3–15.0)
	2013	67 (33–101)	5043 (3861–6225)	2424 (1391–3457)	1.4 (0.5–2.3)	2.9 (0.7–5.1)
Prey remains (nests)	2011	277 (118–436)	3727 (2400–5053)	2653 (1368–3937)	7.4 (2.4–12.4)	10.4 (2.6–18.3)
	2012	150 (75–226)	3059 (2024–4094)	1697 (713–2681)	4.9 (1.9–7.9)	8.8 (2.1–15.6)
	2013	113 (62–165)	5043 (3861–6225)	2424 (1391–3457)	2.2 (1.1–3.4)	4.7 (1.8–7.6)
Pellets (nests)	2011	277 (123–431)	3727 (2400–5053)	2653 (1368–3937)	7.4 (2.5–12.3)	10.4 (2.7–18.1)
	2012	184 (70–297)	3059 (2024–4094)	1697 (713–2681)	6.0 (1.8–10.2)	10.8 (1.6–20.0)
	2013	50 (19–82)	5043 (3861–6225)	2424 (1391–3457)	1.0 (0.3–1.7)	2.1 (0.5–3.6)

Looking at consumption as a percentage of losses for adult grouse (Table 5), during the breeding period we found that, in 2011 and 2012, the uncertainties were so large that the confidence interval included 0 and 100% (excluding 2011 camera estimates where no grouse were recorded). In 2013, estimates ranged from 13% to 29% with confidence interval (CI) extremes of 2 to 53. For grouse chick losses (Table 6), estimate ranges were 0–10% in 2011 (CI extremes 0–18%), 9–11% (2–20%) in 2012 and 2–5% (1–8%) in 2013.

From pellet-based models we estimated that in winter 2013/14, buzzards consumed 400 grouse, equivalent to 11% (4–18%) of grouse present in July 2013, or 33% (8–57%) of the estimated mortality that winter. Corresponding estimates for winter 2014/15 were 242 grouse, equivalent to 7% (2–11%) of those present in July 2014, or 14% (3–25%) of those that died over-winter (Table 5).

## Discussion

Our grouse consumption models estimated that the average breeding buzzard pair plus their chicks consumed between zero and five adult grouse, together with between zero and six grouse chicks, in each breeding season, with values varying between-years and in relation to estimation method. These values are lower than those for a pair of peregrines in the same study area approximately 20 years earlier, which Redpath & Thirgood [56] estimated would kill 13–35 grouse (adults and young) in a breeding season, or for a pair of harriers, which would kill 89–141 grouse chicks [57]. The latter however was considerably less than the 255 grouse chicks estimated by Picozzi [58] for a grouse moor in north-east Scotland. When averaged across diet assessment methods, we estimated that, collectively, the buzzards foraging on our study site could remove 2–5% of grouse chicks hatched, which is comparable to the 0–6% of grouse chicks removed by hen harriers in the presence of diversionary feeding at our study site [29]. Therefore, it is evident that relative to each breeding pair of peregrines and hen harriers in the absence of diversionary feeding, each buzzard pair in this study could have had only a small impact on grouse. However, the number of breeding buzzards in our study years was approximately three-fold higher than peregrine and harrier numbers combined [59], and indices from systematic observations of foraging raptors during the winter were on average 15-times higher for buzzards than for peregrines and harriers combined [60]. Studies attempting to estimate either prey consumption or energetic requirements will be subject to method-based sources of bias and uncertainty [8,19]. Moreover, raptor diet can vary both temporally and spatially in relation to habitat, prey availability and local conditions [4,10,56,61]. By measuring buzzard diet over three breeding and two non-breeding seasons and by using up to three methods, we show not only between-season and between-year variation in diet, but also between-method variation in diet estimation. Diet estimation using prey remains suggested higher grouse consumption by buzzards than when using nest camera and pellets, reflecting already described biases [31,62]. Conversely, direct observations using nest cameras may miss the relatively few deliveries of large birds, which have a proportionately large contribution to total prey biomass. This is highlighted by results from 2011, when cameras recorded no deliveries of grouse, but grouse remains were found both at the nests and in the pellets of the same buzzard pairs.

### Caveats and assumptions

Our estimates of the impact of buzzards on grouse need to be treated with caution for several reasons. Firstly, since buzzards are known to scavenge carcasses, we do not know how many of the grouse recorded in buzzard diet were killed by them or merely scavenged [18,42]. Secondly, we do not know the degree to which buzzard predation on grouse was additive to other causes of grouse mortality [55]. Also, when considering impact levels on grouse chicks, insufficient information was available on age of grouse chicks when consumed. Furthermore, since buzzards, both at Langholm and elsewhere, ate known predators of grouse and their eggs and chicks, such as crows and mustelids [51,63], this may have helped offset the direct impacts of buzzards on grouse. However, predator impact may still be high enough to drive populations to localised extinction in situations where this compensatory predation occurs [10]. Testing these key assumptions was beyond the scope of our study but will be crucial to improving robustness of our estimates.

Inherent in our estimates of buzzard diet is the assumption that our measures were representative of all buzzards present at the study site. Despite sampling most breeding pairs, we did not consider diet of non-breeders in the breeding season, whose hunting efficiency and prey spectrum may have differed from breeders [64]. Given the predicted high proportion of non-breeders in the population [36], this could have potentially altered model outputs, but in an unknown direction relative to the number of grouse consumed.

Winter diet was measured only from pellets, which can overestimate indigestible prey such as hard-bodied insects relative to soft-bodied prey or items where only flesh is eaten [30,31,65]. Our feeding trial using captive buzzards confirmed that diet estimates from pellets alone may underestimate grouse presence by approximately half, and so we adjusted biomass proportions for the purpose of grouse consumption models.

Estimates of buzzard numbers also had a large effect on model outputs. We assumed a breeding rate of 35.5% [36], but since publication of those data, buzzards have increased in abundance and range in Britain [11] and breeding rates may now differ. Our estimation of numbers does not consider movements on and off the site. If it did, it would probably reduce the ratio of tagged to un-tagged birds and hence inflate estimates of numbers present.

### Management implications

Between 2008 and 2015 approximately UK£225,000 (circa 250,000 euros) were invested annually into employing and equipping five gamekeepers at our study site, which does not include the additional costs of habitat restoration or infrastructural improvement [59]. Despite this, grouse did not recover sufficiently to recommence driven shooting at a predicted economic return of £150 per brace (two) for shot grouse [21] and hence continued management was deemed economically unviable. The years of this study coincided with the period when grouse densities were at their highest during the ten-year partnership project operating on our study site (mean July grouse density in 2011–2015 = 95 grouse/km^2^, range: 73–123; [66]), which is lower than the 133 grouse/km^2^ threshold considered most likely for driven shooting to occur [67]. It is possible that grouse consumption by buzzards in these years contributed to preventing grouse densities exceeding this threshold, although this depends on the extent to which buzzards killed the grouse they ate, as well as any compensatory effects of buzzard predation on other grouse predators.

Buzzard consumption of breeding grouse varied between years, despite no evidence of between-year variation in buzzard abundance or productivity. This suggests that annual grouse consumption varied not in relation to buzzard abundance, as has been shown for harriers on the same site [56], but instead in relation to the proportion of grouse in buzzard diet. This observation is consistent with previous research at Langholm, which suggested that higher vole densities resulted in increased buzzard foraging on vole-rich moorland habitats, where they had higher grouse encounter rates, and consequently ate more grouse [61]. Numerous studies have demonstrated the importance of this type of incidental predation, whereby predation rates can be driven by spatial distributions and temporal fluctuations in preferred prey [35,68–70], including in buzzards [71]. Thus buzzard predation rates on grouse may be greater during high vole density years, especially on moors such as Langholm where a high grass-to-heather ratio may naturally favour high vole abundance [72]. As such, efforts to reduce predation on a valued prey resource (such as grouse) may benefit from management practices that reduce the attractiveness to predators of the habitats that contain these prey. On our study site, it may be that increasing heather cover at the expense of grassland will benefit grouse by, firstly providing more primary grouse habitat, and secondly reduce suitability to voles thus reducing incidental predation by buzzards [61]. Equally, management that seeks to attract buzzards away from grouse rich habitats, such as encouraging vole and rabbit-rich grassland habitats or providing diversionary food away from the heather moor [29,73] could also prove effective at reducing grouse predation. The key assumptions highlighted within this study, together with the associated wide confidence intervals resulting from the multiplicity of estimates and the variability in parameters used to produce them, need to be cautiously considered prior to implementation of any mitigating management. Nevertheless, our results suggest that management practices seeking to reduce buzzard foraging in grouse habitats, such as those outlined above, could prove beneficial to grouse recovery efforts on our study site.

Estimating impacts of raptor predation on gamebirds is a contentious subject [74] which is difficult to address in the absence of experimental approaches [7]. Our study demonstrates how a combination of direct and indirect observations of predator consumption of prey, presented within a bioenergetics framework, can help our understanding of the possible impacts of predators on prey groups.

## Supporting information

S1 Table Results from captive buzzard feeding trial. (XLSX)
